# Sensory Quality of Macadamia Nut Butter: Effects of Kernel Grade and Maturity

**DOI:** 10.1002/fsn3.70773

**Published:** 2025-08-08

**Authors:** Tarryn Amber Ohlsson, Marieta van der Rijst, Jeannine Marais

**Affiliations:** ^1^ Department of Food Science Stellenbosch University Matieland South Africa; ^2^ Biometry Unit Agricultural Research Council, Infruitec‐Nietvoorbij Stellenbosch South Africa

**Keywords:** descriptive analysis, kernal quality, *Macadamia integrifolia*, macadamia nut butter, style, tree nut

## Abstract

Macadamia nut butter is a value‐added product, promoting lower‐grade nuts to premium products. The commercial grade of macadamia nuts includes the kernel quality (i.e., maturity and oil content) and style (i.e., size). However, whether immature nuts are suitable for nut butter production remains under‐explored. The study aimed to determine the influence of macadamia nut qualities and styles on the sensory profile of macadamia nut butter. Individual butters from two high oil, mature qualities (Style 1, Style 8) and two low oil, immature qualities (Style 2, Style 4) were compared to a commercial control. Descriptive sensory analysis was performed with an experienced panel (*n* = 13), a combination of ballot and consensus training, and reference standards. A sensory lexicon of 40 descriptors was developed, where 35 attributes differed (*p* < 0.050) between treatments. Principal component analysis (70.42% explained variance) confirmed variability in the sensory profiles of commercial control batches. High oil, mature kernels produced more replicable butter quality. Low oil, immature kernels produced inconsistent quality butters, with higher association with positive attributes (e.g., *nutty* aroma/flavor), but also with off‐notes (e.g., *fruity* aroma/flavor). The results suggest that kernel quality (maturity and oil content), rather than style, has the greatest influence on the final sensory quality of nut butter. These findings provide practical guidance for manufacturers in the procurement of macadamia kernel grades for butter production and the implications on product sensory quality. Further investigation is needed to customize roasting and grinding times based on kernel quality, which may improve product consistency.

## Graphical abstract

This study investigated how macadamia nut quality (maturity and oil content) and style (size) influence the sensory profile of macadamia nut butter. Descriptive sensory analysis showed that kernel quality, rather than style, most strongly affected butter characteristics, with mature, high‐oil nuts producing more consistent quality. Immature nuts yielded both desirable and off‐flavor attributes, indicating the need for tailored processing to optimize product quality.

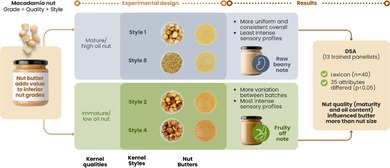

Graphical abstract, for sensory quality of nut butter produced from different commercial macadamia grades, with sample photos belonging to the authors. The jar stock image, broken macadamia nut image, and icons were sourced from canva.com.

## 1 Introduction

Macadamia nuts ( *Macadamia integrifolia* seeds) are one of the most profitable crops produced in subtropical climates and are very popular with consumers interested in foods marketed as ‘health’ (Wall 2013; Alam and Grose 2022; Bouarakia et al. 2023). The global demand for macadamia nuts is rising with an increased need for healthy, great‐tasting snacks (Shuai et al. 2024; Swinehart and Feng 2023). However, the production and processing of macadamia nuts in leading producer countries, such as China, are unable to match the pace of consumption and demand (Shuai et al. 2023). Additionally, poor kernel quality (e.g., immature, molding, or insect damage) threatens production with estimated losses of *ca*. 4%. This may be a severe underestimate, as damaged kernels may not reach the packing house or processor, excluding them from being counted (Bouarakia et al. 2023). Evaluating the potential use of lower‐quality edible kernels, like immature macadamia nuts, can enhance their value in macadamia products such as nut butter.

In general, nuts can be processed into nut butter (≥ 90% nut content), which is a convenient processed nut product, diversifying the offerings available for the nut industry (USDA 2019; Zuza et al., n.d.). The nut industry may also benefit from the value‐adding potential of nut butters, particularly in elevating lower‐grade nuts (Shakerardekani et al. 2013). Consumers may benefit from increased functional food product offerings made available through nut butters, which align with trends in vegetarian and vegan diets (Vene et al. 2025). More practically, smooth nut butters allow consumers with dental issues the opportunity to enjoy nuts (Neale et al. 2020; Yong et al. 2017). Since nut maturity affects final product quality (Shakerardekani et al. 2013; Wall 2013), assessing different macadamia grades for nut butter is essential.

The World Macadamia Organization (WMO) defines three grades of macadamia nuts: premium, commercial, and reject. Premium kernels are fully mature and free of defects, whereas commercial kernels have slight surface imperfections or immaturity (WMO 2023). Rejected kernels have more serious defects. Macadamia nuts are further classified into Style groups based on kernel size. There is a range of Styles, from whole nuts (Style 0) to fine pieces (Style 8) (Wall 2013). Thus, the grade of a macadamia kernel can describe both its quality and size.

Higher macadamia nut oil content is an indicator of macadamia kernel maturity and quality (Wall & Gentry, 2007; Wall 2013). During nut maturation, hexose sugars are transformed into fatty acids (Hu et al. 2019; Wall 2013). This results in immature nuts having higher reducing sugars (such as sucrose), facilitating increased nonenzymatic browning during roasting and lower oil content overall (Wall 2013). More mature macadamia kernels, on the other hand, have lower hexose sugar and higher oil contents (Gama et al. 2020).

There are various quality assurance testing methods to analyze the physicochemical properties of macadamia nut butter (Shuai et al. 2024). However, whether sensory quality differences in macadamia nut butter exist due to kernel style and quality remains underexplored in the literature. This knowledge gap may have led to inconsistency in the current macadamia nut butter offering (e.g., variances in color or flavor). This indicates that analytical sensory analysis of macadamia nut butter varieties is needed to determine and standardize the sensory quality of the product. Descriptive sensory analysis (DSA) is a promising technique for investigating the quality of nut butters. This method has been used to profile the sensory quality of cashew butter (Lima et al. 2012), peanut butter (Ding et al. 2024; Hathorn and Sanders 2012; Sanders et al. 2014), and walnut butter (Leahu et al. 2022). Therefore, the aim of the present study was to analyze the sensory quality of various macadamia nut butter varieties made from different nut styles and qualities, using DSA with trained panelists. It is hypothesized that kernel maturity and oil content would significantly influence the sensory profile of macadamia nut butter. Additionally, selected physicochemical analyses were conducted as quality indicators, including viscosity, water activity, surface color, oil separation, degree of uniformity, and total fat content.

## 2 Materials and Methods

### 2.1 Experimental Design

This study included five treatments of macadamia nut butter (Table 1). The first treatment was a commercial control (a commercially available macadamia nut butter supplied by a nut butter manufacturer). Additionally, four experimental treatments were included in the study, each prepared from four different macadamia nut styles (Styles 1, 2, 4, and 8) (Wall 2013). The raw nut supplier classified the macadamia kernels from style 2 as ‘low oil’ kernels and those from style 4 as ‘immature’. Thus, the treatments were classified as two high oil, mature qualities (Style 1, Style 8) and two low oil, immature qualities (Style 2, Style 4). For each experimental treatment (*n* = 5), eight independent batches of macadamia nut kernels (i.e., individually sealed bags, serving as independent statistical replicates; *N* = 40) were included in the study.

**TABLE 1:** Description of treatments, including the macadamia nut style and grinding time associated with each treatment.

Treatment	Treatment description	Roasted macadamia kernel/piece appearance	Total grinding time per 550 g grinding set^†^
Commercial control	Commercial control	N/A	Unknown
Style 1	95% whole macadamia kernels (> 13 mm), and 5% halves and pieces (> 13 mm) (UNECE 2011).	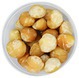	35 s
Style 2	70% whole (16–20 mm) and 30% half (> 13 mm) (UNECE 2011). Labeled as low oil by the supplier.	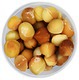	150 s
Style 4	Medium/large half kernels (6–13 mm in size) (UNECE 2011). Labeled as immature by the supplier.	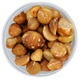	80 s
Style 8	Pieces that are < 3 mm in size (UNECE 2011).	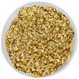	42 s

^†^ Grinding time had to be adapted per Style, as treatments with higher oil content were easier to blend and required less grinding time. Lower oil treatments required more time to blend, as they produced a thicker paste before homogenizing (refer to Figures S2–S5). However, the grinding time was consistent within a treatment group.

### 2.2 Sample Preparation

The small‐scale production of four treatment batches closely resembled the commercial practices of the supplier of the control sample, as depicted in Figure S1. All raw macadamia nuts ( *Macadamia integrifolia* ) were obtained from three macadamia nut primary processors (supplied by > 650 growers), based in the Mpumalanga and KwaZulu‐Natal provinces of South Africa. The raw macadamia nuts were packed in 11.34 kg (or 25 lb) vacuum‐sealed bags by the suppliers and stored at ambient temperature (15°C–35°C) prior to roasting.

All experimental treatment samples (*n* = 32) were roasted at 180°C for 20 min in a rotary rack oven (Macadams Baking Systems, Cape Town, South Africa). Following roasting, large fans were used to cool the roasted kernels for *ca*. 15 min. Once cooled, a representative sample (1.1 kg) of roasted kernels could be taken from the tray to be temporarily stored in 2.5 L plastic buckets.

The roasted nut batches (1.1 kg) were blended at 3000 rpm (Robot Coupe Blixer 3, 3.7 L) while stirring continuously. Although the grinding speed was 3000 rpm for each treatment, the grinding time varied per treatment (Table 1). Notably, lower oil and immature varieties required more time to homogenize (Figures S2–S5). For consistency, the first experimental batch was blended until the viscosity matched that of the Commercial control when dripping a teaspoon back into the blender. The grinding time for the treatment was noted, and the subsequent batches were blended for this designated grinding time. Once blended, all macadamia nut butter batches were refrigerated (4°C ± 1°C) in 2.5 L plastic buckets. The production of the Commercial control is not discussed for confidentiality purposes.

### 2.3 Physicochemical Measurements

#### 2.3.1 Viscosity

The viscosity was measured in triplicate for each treatment batch (*N* = 40). A Perten Rapid Visco Analyzer (RVA 4500) coupled to an external cooling system (SMC refrigerated bath) was used. A 30 g sample of each treatment was decanted into the aluminum cannister, and the batch was measured at 21°C ± 1°C, equivalent to the serving temperature during sensory evaluation (section 2.4). The temperature was controlled with ThermoCline for Windows (TCW) software (version 3.1). The plastic paddle was set to rotate at a speed of 160 rpm (Chen et al. 2022) for 5 min. The results were captured using TCW software and exported to Microsoft Excel (Version 2402) for data preparation. Data cleaning involved removing the first 3 min of results to ensure the samples had acclimated to 21°C ± 1°C. Thus, the results included 181 s to 300 s time points (i.e., 2 min). Samples were pre‐conditioned by homogenization prior to viscosity measurements to ensure consistency across batches.

#### 2.3.2 Oil Separation

Oil separation within the macadamia nut butter samples was measured in triplicate for all treatment batches (*N* = 40) through a centrifugation method adapted from Shuai et al. 2023. A 1.8 g sample of macadamia nut butter was transferred into a polypropylene 2 mL microcentrifuge tube. Twenty‐four sample tubes were placed into the fixed‐angle Neofuge 13 centrifuge (Heal Force Bio‐Meditech Holdings Group Nison Instrument, Shanghai). These samples were centrifuged at 16217 rcf (relative centrifugal force, or 13,300 rpm) for 60 min at ambient temperature (22°C ± 2°C), which was selected to replicate typical storage conditions without inducing phase transitions in the macadamia nut butter. Thereafter, the tubes were inverted, and the oil could be poured out of the tube. The remaining oil could be carefully blotted with a clean paper towel (Shuai et al. 2023). The amount of oil that was separated was calculated using Equation (1).

(1) $$\text{Oil separation}\;\left(\mathrm{OS}\right)=100\left(M-m\right)/\left(M-m_{\text{tube}}\right)$$

Equation (1) To calculate the percentage of separated oil from macadamia nut butter (adapted from Shuai et al. 2023).

M = mass of microcentrifuge tube with oil (g).

m = mass of microcentrifuge tube without oil (g).

m_tube_ = mass of microcentrifuge tube = 1.01 g.

#### 2.3.3 Color

The color was measured in triplicate for all treatment batches (*N* = 40) using a CIELab colorimeter (portable BYK spectro guide 45/0 gloss). The color measurement was taken at a 90° angle to the sample to minimize reflection artifacts and ensure consistent readings across batches. Each sample was spread evenly in a petri dish before measurement to maintain uniform surface exposure. The hue angle (h°; Shuai et al. 2024), chroma (C*; Shuai et al. 2024), and browning index (BI) were derived from the L*, a*, and b* values, with the BI calculated using Equation (2).

(2) $$\text{Browning Index}\;\left(\mathrm{BI}\right)=100\;\left(x-0.31\right)/0.17$$

where *x* = (a + 1.75 L*)/(5.64 L* + a*−3.012b*).

Equation (2) To calculate the browning index (BI) based on L*, a*, and b* color values (adapted from Yoon et al. 2024).

*x* = color ratio.

a* = coordinate position on the green‐red axis.

b* = coordinate position on the blue‐yellow axis.

L* = coordinate position on the lightness axis.

#### 2.3.4 Uniformity

The uniformity among the treatment batches (*N* = 40) was observed with a NIKON D5600 camera equipped with a 35 mm focal lens selected to ensure a balanced field of view for accurate image capture. A single sample of each treatment batch was decanted into 70 mm diameter plastic petri dish lids and then placed into the assessment box (Puluz Dual LED Photo Softbox Studio Box Kit, 40 × 40 × 40 cm). The light box was fitted with a silver reflective interior, and a white, non‐gloss PVC backdrop was added to minimize any light reflection from the glossy macadamia nut butter samples and two D55 LED light strips (2 × 1200 lm and a light temperature of 5500 K). A photo was taken at a 90° angle to the sample. Standardized camera settings were applied: autofocus point selection was manually set to the center, with an f‐stop of f/5.6, an exposure time of 1/1600 s, an ISO speed of ISO‐400, and exposure bias set to 0 step. The maximum aperture was 4.4, and metering was adjusted using a pattern mode. No flash, additional lenses, or filters were used, and images were saved in JPG format with normal contrast and auto white balance to maintain consistency across all photographs.

A photographic standard (Figure S6) was placed next to each sample for photography. This color standard was used as an aid in post‐photographic editing. A gray reference, for example, is beneficial in improving the standardization of digital images and has been noted to improve this in prosthetic dentistry (Sampaio et al. 2019). Each sample photo was adjusted to the same brightness balance, based on the gray standard. Contrast was added where color adjustments were needed until the blue and magenta were balanced based on the color standard. The extent of post‐photographic editing differed slightly for each sample photo.

#### 2.3.5 Total Fat Content

The measurement of the total fat content of the nut butters was done in duplicate for all treatment batches (*N* = 40). The protocol for the quantification of fat in butter using the Mojonnier method (adapted from Marshall 1992) was used to quantify the total fat of macadamia nut butter. Two extractions with ethanol, diethyl ether, and petroleum ether were performed. The solvents were added sequentially to facilitate phase separation and optimize fat recovery efficiency. However, an additional 10 mL of ethanol was used in each extraction to account for an emulsion that formed, allowing clearer separation between the organic and aqueous layers. The fat percentage of each treatment batch was calculated (Moneeb et al. 2021).

### 2.4 Descriptive Sensory Analysis (DSA)

#### 2.4.1 Reference Standards

A set comprising 32 food and chemical reference standards was prepared to facilitate concept formation during panel training for descriptive sensory analysis (Table 2). Some of the references, such as the expired nuts and nut butter, were only used as aroma references.

**TABLE 2:** Reference standards used for panel training for descriptive sensory analysis, including their related sensory attributes, scores, serving containers, and preparation methods.

Reference standards	Image	Description	Sensory attribute	Score^1^	Serving container and quantity	Preparation
Color gradient chart	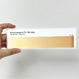	Customized gradient color chart of light to dark shades that cover the range of darker to lighter macadamia butter in the treatment batches (*N* = 40)	*Brown color* intensity	0–100	N/A	Reference colors were matched to Pantone color swatches to establish standardized visual anchors. The lightest butters (Pantone 468 C) and darker nut butters (Pantone 466 C) were included. These were further extended to lighter shades (Pantone 7499 C) and darker shades (Pantone 157 C) using CorelDRAW x7, 2014. The finalized color charts were printed on an HP1510 printer and laminated for durability
Melted unsalted butter	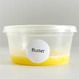	Melted unsalted butter	*Buttery* aroma	100	15 mL served in a plastic container (75 mL)	The unsalted butter (Woolworths Food, South Africa) was melted in a microwave (2450 MHz, 230 V, 1000 W) for 1 min
Raw macadamia paste	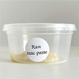	Blended raw macadamia nuts	*Brown color* *Roasted nut* aroma *Nutty sweet* aroma *Raw beany* aroma *Nutty* aroma *Dairy sour* aroma *Roasted nut* flavor *Raw beany* flavor *Nutty sweet* flavor *Nutty* flavor *Graininess*	10 0 15–20 20–40 30 30 0 30 40 50 80	5 g served in a plastic container (75 mL) with a lid and a spoon	Raw macadamia nut kernels (Woolworths Food, South Africa) were blended for 1 min using a Milex Nutri1000 (1000 W, 20000 rpm)
Raw tree nut paste	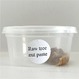	Blended raw luxury tree nut kernels (almonds (25%), Brazil nuts (25%), cashew nuts (25%), hazelnuts (25%))	*Nutty* aroma *Raw beany* aroma *Raw beany* flavor	20 60 70	5 g served in a plastic container (75 mL) with a lid and a spoon	Raw luxury tree nut kernels (Woolworths Food, South Africa) were homogenized—blended for 1 min using Milex Nutri1000 (1000 W, 20000 rpm)
Roasted macadamia paste	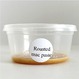	Raw macadamia nuts roasted and blended	*Brown color* *Nutty sweet* aroma *Buttery* aroma *Nutty* aroma *Roasted nut* aroma *Raw beany* flavor *Buttery* flavor *Nutty sweet* flavor *Nutty* flavor *Roasted nut* flavor *Graininess*	85 50 50 80 90 0 60 60 80 90 60	5 g served in a plastic container (75 mL) with a lid and a spoon	Raw macadamia kernels (Woolworths Food, South Africa) were roasted at fan speed 1 in a Hobart convection oven (CSD 1012E, France) at 180°C for 4 min and then cooled for 10 min. Roasted macadamias were then homogenized—blended for 1 min using Milex Nutri1000 (1000 W, 20000 rpm)
Nestlé caramel treat	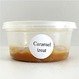	Caramel treat (full cream milk, sugar, lactose powder)	*Caramel* aroma *Caramel* flavor	100 100	15 g served in a plastic container (75 mL) with a lid and a spoon	Caramel treat (Nestlé, South Africa) was served as is, with no additional preparation
Cultured Cream Crème Fraîche	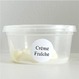	Cultured Cream Crème Fraîche (cream, milk, skimmed milk powder, stabilizer, cheese culture)	*Dairy sour* aroma *Sour* taste	50 50	15 g served in a plastic container (75 mL) with a lid and a spoon	Cultured cream crème fraiche (Woolworths Food, South Africa) was served as is, with no additional preparation
Borneol solution (1% PG)	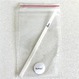	Borneol chemical reference standard (1% PG)	*Earthy* aroma	100	Dipped smelling strip served in a zip‐lock plastic bag	Smelling strips dipped into Borneol 1% propylene glycol (PG) (MANE, South Africa) and sealed in a plastic zip lock bag
1% raw honey solution	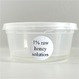	100% Raw honey	*Honey* aroma (1) *Honey* flavor (1)	30 10	14 g served in a plastic container (75 mL) with a lid and a spoon	Raw honey (Woolworths Food, South Africa), was prepared in a 1% solution using boiled water and allowed to cool
2% raw honey solution	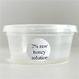	100% Raw honey	*Honey* aroma (2) *Honey* flavor (2)	50 40	14 g served in a plastic container (75 mL) with a lid and a spoon	Raw honey (Woolworths Food, South Africa) was prepared in a 2% solution using boiled water and allowed to cool
0.2% Marmite solution	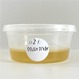	Marmite (yeast extract, water, salt, flavoring, spices (allspice))	*Savory* aroma *Savory* flavor	40 20	50 mL served in a plastic container (75 mL) with a lid and a spoon	Marmite (Pioneer Foods, South Africa) was prepared in a 1% solution using boiled water. Once cooled, filtered tap water was added to achieve a 0.2% dilution
0.2% vanilla solution	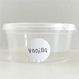	0.2% solution of vanilla paste (Mineral water, glucose, humectant, ethanol, vanilla (6%), stabilizer)	*Vanilla* aroma	20	50 mL served in a plastic container (75 ml)	Vanilla paste (Woolworths Food, South Africa) was prepared in a 1% solution using boiling water. Once cooled, filtered tap water was added to achieve a 0.2% dilution
Macadamia oil^2^	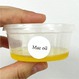	100% Macadamia oil (cold pressed)	*Rancid* aroma *Oily* flavor *Rancid* flavor *Oily mouthcoating*	0 80 0 40	10 g served in a plastic container (75 ml) with a lid and a spoon	Macadamia oil (Woolworths Food, South Africa) was served as is, with no additional preparation
Expired macadamia nut kernels	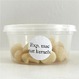	Raw, expired Style 5 macadamia nut kernels (BB August 2023, i.e., 4 months expired)	*Rancid* aroma	80	15 g served in a plastic container (75 ml)	Raw, expired Style 5 macadamia nut kernels (Khuvuka, South Africa) were served as is, with no additional preparation
Raw, expired macadamia butter	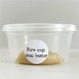	Butter made from raw, expired Style 5 macadamia nut kernels (BB August 2023, i.e., 4 months expired)	*Glossiness* *Visual thickness* *Rancid* aroma	80 90 40	5 g served in a plastic container (75 ml) with a lid and a spoon	Macadamia butter produced from expired kernels (Khuvuka, South Africa) was blended for 1 min 10 s using a Milex Nutri1000 (1000 W, 20000 rpm)
Raw, expired macadamia butter with 10% macadamia oil	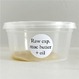	Butter made from raw expired macadamia kernels (BB August 2023, i.e., 4 months expired) with an additional 10% of 100% macadamia oil (cold‐pressed)	*Glossiness* *Visual thickness*	90 70	15 g served in a plastic container (75 ml) with a lid and a spoon	Macadamia butter produced from expired kernels (Khuvuka, South Africa) was blended for 1 min 10 s using a Milex Nutri1000 (1000 W, 20000 rpm). Subsequently, 10% by mass of 100% macadamia oil (Woolworths Food, South Africa) was added and blended for an additional 1 min
Smooth peanut butter	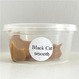	Smooth peanut butter [peanuts (91% minimum), cane sugar, stabilizers (E471), salt]	*Visual graininess* *Sweet* taste *Graininess* *Oily mouthcoating* *Stickiness* *Thickness*	0 40 0 0 80 90	15 g served in a plastic container (75 mL) with a lid and a spoon	Black Cat smooth peanut butter (Tiger Brands, South Africa) was served as is, with no additional preparation
Raw peanuts	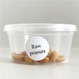	Raw peanuts (without skins)	*Raw beany* flavor	20	15 g served in a plastic container (75 ml) with a lid and a spoon	Raw peanuts without skins (L & C Messaris Bros. MRG, South Africa) were served as is, with no additional preparation
Roasted peanuts	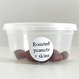	Redskin peanuts with skins (plain roast)	*Earthy* flavor	60	15 g served in a plastic container (75 ml) with a lid and a spoon	Redskin peanuts with skins (JAV Trading t/a Eden, South Africa) were served as is, with no additional preparation
Dark roasted peanuts	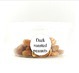	Raw peanuts without skins	*Burnt* flavor *Bitter* taste	70 30	7.5 g served in a plastic container (75 mL) with a lid and a spoon	Raw peanuts (L & C Messaris Bros. MRG, South Africa) were roasted in a Hobart convection oven (CSD 1012E, France) (fan speed 1) at 180°C for 16 min
Burnt toast	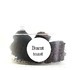	Burnt white sandwich bread (white bread wheat flour, water, yeast, salt, may contain an acidity regulator seasonally, soybean flour, emulsifiers (vegetable origin), non‐hydrogenated vegetable fat (palm fruit), preservative (calcium propionate), flavor enhancer, flour improver, enzymes (non‐animal origin), mineral salts (electrolytic iron and zinc oxide) and vitamins (vitamins B3, B6, B1, B2, A and folic acid))	*Burnt* aroma	100	15 g served in a plastic container (75 ml) with a lid and a spoon	White sandwich bread (Woolworths Food, South Africa) was baked in a Hobart convection oven (CSD 1012E, France) (fan speed 1) at 120°C for 45 min, followed by an additional 12 min at 200°C
Condensed milk	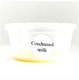	Full cream sweetened condensed milk (milk, sugar, lactose powder)	*Stickiness* *Thickness*	10 20	15 g served in a plastic container (75 ml) with a lid and a spoon	Full cream, sweetened condensed milk (Nestlé, South Africa) was served as is, with no additional preparation
Baked banana with peel	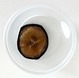	Baked banana with peel	*Fruity off* aroma	100	A slice of banana (1 cm) served in a plastic container (75 ml) with a lid	Bananas (Woolworths Food, South Africa) with peel were sliced into 16 even pieces (*ca*. 1 cm thick) and then placed in a glass ramekin. These were baked in Hobart convection oven (CSD 1012E, France) (fan speed 1) at 180°C for 5 min and allowed to cool
Banana chips	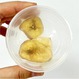	Honey‐dipped banana chips (banana, vegetable oil, coconut fruit, sugar, honey, banana flavoring)	*Fruity off* flavor	50	Two banana chips served in a plastic container (75 ml) with a lid	Honey‐dipped banana chips (JAV Trading t/a Eden, South Africa) were served as is, with no additional preparation
2% sucrose solution		2% sucrose solution	*Sweet* taste	20	50 mL served in a white wine glass with a cover	2% sucrose (Tongaat Huletts Sugar, Limited, South Africa) solution in water
5% sucrose solution		5% sucrose solution	*Sweet* taste	50	50 mL served in a white wine glass with a cover	5% sucrose (Tongaat Huletts Sugar, Limited, South Africa) solution in water
0.2% NaCl solution		0.2% sodium chloride solution	*Salty* taste	20	50 mL served in a white wine glass with a cover	0.2% sodium chloride (Cerebos, South Africa) solution in water
0.35% NaCl solution		0.35% sodium chloride solution	*Salty* taste	40	50 mL served in a white wine glass with a cover	0.35% sodium chloride (Cerebos, South Africa) solution in water
0.05% citric acid solution		0.05% citric acid solution	*Sour* taste	30	50 mL served in a white wine glass with a cover	0.05% citric acid (Robertsons, Unilever, South Africa) solution in water
0.08% citric acid solution		0.08% citric acid solution	*Sour* taste	55	50 mL served in a white wine glass with a cover	0.08% citric acid (Robertsons, Unilever, South Africa) solution in water
0.05% caffeine solution		0.05% caffeine solution	*Bitter* taste	30	50 mL served in a white wine glass with a cover	0.05% caffeine solution in water
0.08% caffeine solution		0.08% caffeine solution	*Bitter* taste	60	50 mL served in a white wine glass with a cover	0.08% caffeine solution in water

^1^ Score intensities were rated on a 100‐point line scale with anchor words at the 0 (None) to 100 (Prominent) ends, except for *Brown color* (where 0 = light, and 100 = dark), *Visual thickness* (0 = thin, 100 = thick), *Visual graininess* (0 = none, 100 = abundant), *Uniformity of granules* (0 = not uniform (irregular), 100 = uniform).

^2^ Macadamia oil aroma was noted to resemble the aroma of hay.

#### 2.4.2 Panel Training

Thirteen sensory panelists (12 female and 1 male, 23–72 years old) were recruited and screened for adequate sensory acuity according to the guidelines by Rogers (2018). These panelists had several years of experience in participating in the descriptive sensory analysis (DSA) methodology and were deemed experienced sensory panelists. The panelists completed a written consent form to participate in this study, which was approved by the Social, Behavioral, and Educational Research Ethics Committee (REC: SBE‐2023‐28107) of Stellenbosch University, South Africa.

The panel training for DSA followed a combination of ballot and consensus training (Lawless and Heymann 2010). As there was not yet an existing lexicon for macadamia nut butter, a ballot was compiled using existing literature. This included almond butter, cashew butter, hazelnut butter, peanut butter, pistachio butter, as well as nutty‐associated attributes in general (Cadena et al. 2012; Eker et al. 2023; Gama and Adhikari 2019; Larssen et al. 2018; Lima et al. 2012; Zhou et al. 2023). Thereafter, consensus training with the initial ballot streamlined the process of generating a lexicon.

Panel training occurred over 5 days, with two 1 h training sessions per day (i.e., 10 h of training in total). This training period was deemed adequate as all panelists reached consensus on the comprehensive list of product attributes and their descriptors. Additionally, they demonstrated consistent calibration in scale use and scoring, applying the full sensory lexicon reliably and with consensus. A 15–20 min break was given between sessions. At each training session, the panelists were presented with all reference standards (Table 2) and five labeled containers (one replication per treatment group). All treatment samples were stirred thoroughly before being decanted from the storage containers into the serving containers (75 mL plastic tubs with lids) and were served at 21°C ± 1°C.

Carrot batons (Woolworths Food, South Africa) were used as initial palate cleansers (Wagener and Kerr 2018) alongside filtered potable tap water from a municipal supply (safe to drink). However, the carrot batons were deemed ineffective in palate cleansing due to their sweetness. Cucumber slices (Woolworths Food, South Africa) (Nakitto et al. 2022) were considered more appropriate palate cleansers for macadamia nut butter. Sparkling water (Woolworths Food, South Africa) (Wagener and Kerr 2018) was deemed more effective in palate cleansing than still water, perhaps due to carbonation. Nonetheless, the panelists were provided with both still and sparkling water.

#### 2.4.3 Intensity Rating

Following the panel training, sensory evaluation took place over 4 days. Each day included two sessions, testing two batches per session (*n* = 8 batches per treatment). Panelists scored the intensities of the various sensory attributes from the developed lexicon (*N* = 40; Table 3) on a 100‐point unstructured line scale. Each panelist was allocated to an individual tasting booth with controlled white lighting. The temperature of the testing facility was controlled at 21°C ± 1°C. Communication among panelists was prohibited while evaluating samples.

**TABLE 3:** Sensory attributes included in the macadamia butter lexicon, as determined through descriptive sensory analysis using trained panelists.

Attribute	Description	Reference standards (final scores)^6^
*Appearance*		
Brown color 0 = light; 100 = dark	The intensity of the brown color associated with macadamia nuts^1^, ^2^	Color gradient chart Raw macadamia paste (10) Roasted macadamia paste (85)
Glossiness^7^	The appearance associated with the amount of light reflected by the product surface (higher for oily products)^1^, ^3^	Raw expired macadamia butter (80) Raw expired macadamia butter + oil (90)
Visual thickness 0 = thin; 100 = thick	The perception of product thickness on a teaspoon and the ability of the sample to stay on the spoon when turned vertically and allowed to drip^3^	Raw expired macadamia butter + oil (70) Raw expired macadamia butter (90)
Visual graininess 0 = none; 100 = abundant	The amount of small pieces as seen visually on the back of a coated teaspoon^3^	Black Cat smooth peanut butter (0)
Uniformity of granules 0 = not uniform (irregular); 100 = uniform	The similarity in size of the granules that are seen visually on the back of a coated teaspoon	
*Aroma* ^*7*^		
Nutty aroma	Aromatics associated with unspecific nuts^3^	Raw luxury tree nut paste (20) Raw macadamia paste (30) Roasted macadamia paste (80)
Buttery aroma	Characteristic slightly melted butter (i.e., pasteurized cream) aromatics	Roasted macadamia paste (50) Melted unsalted butter (100)
Roasted nut aroma	Aromatics associated with medium‐roasted macadamia nut kernels^1^, ^2^, ^3^	Raw macadamia paste (0) Roasted macadamia paste (90)
Nutty sweet aroma	Sweet aromatics associated with roasted nuts^1^, ^2^	Raw macadamia paste (15–20)^8^ Roasted macadamia paste (50)
Raw beany aroma	Aromatics associated with uncooked or raw macadamia nuts (slightly vegetative in character)^1^	Raw macadamia paste (20–40)^8^ Raw luxury tree nut paste (60)
Dairy sour aroma	Aromatics associated with dairy‐sour notes (i.e., after adding starter cultures to milk during cheese‐making)^4^	Raw macadamia paste (30) Crème Fraîche (50)
Caramel aroma	Sweet aromatics characteristic of molten sugar or caramel pudding	Nestlé caramel treat (100)
Savory aroma	Aroma associated with a combination of savory, salty, and yeasty notes	0.2% Marmite solution (40)
Rancid aroma	Aromatics associated with rancid fats and oils (including cardboard, painty, and chemical notes)^1^	Macadamia oil (0) Raw expired macadamia butter (40) Expired macadamia nut kernels (80)
Honey aroma	The sweet, light brown, slightly spicy aromatics associated with raw honey	1% raw honey solution (30) 2% raw honey solution (50)
Burnt aroma	Aromatics associated with a very dark roast, burnt starches, or carbohydrates	Burnt toast (100)
Earthy aroma	Aromatics associated with damp soil	Borneol solution (1% PG) (100)
Vanilla aroma	Sweet aromatic associated with cake mix or vanillin^5^	0.2% Vanilla paste solution (20)
Fruity off aroma	Aromatics associated with overripe tropical fruit (associated with banana peel, dried banana, dried pineapple, and/or guava)	Baked banana with peel (100)
*Palate* ^*7*^		
Nutty flavor	Aromatics associated with unspecific nuts^3^	Raw macadamia paste (50) Roasted macadamia paste (80)
Buttery flavor	The intensity of characteristic slightly melted butter (i.e., pasteurized cream) aromatics	Roasted macadamia paste (60)
Roasted nut flavor	Aromatics associated with medium‐roasted macadamia nut kernels^1^, ^2^, ^3^	Raw macadamia paste (0) Roasted macadamia paste (90)
Nutty sweet flavor	Sweet aromatics associated with roasted nuts^1^, ^2^	Raw macadamia paste (40) Roasted macadamia paste (60)
Raw beany flavor	Aromatics associated with uncooked or raw macadamia nuts (slightly vegetative in character)^1^	Roasted macadamia paste (0) Raw peanuts (20) Raw macadamia paste (30) Raw luxury tree nut paste (70)
Caramel flavor	Sweet aromatics characteristic of molten sugar or caramel pudding	Nestlé caramel treat (100)
Savory flavor	Flavor associated with a combination of savory, salty, and yeasty notes	0.2% Marmite solution (20)
Honey flavor	The sweet, light brown, slightly spicy aromatics associated with raw honey	1% raw honey solution (10) 2% raw honey solution (40)
Oily flavor	Aromatics associated with macadamia oil	Macadamia oil (80)
Rancid flavor	Aromatics associated with rancid fats and oils (including cardboard, painty, and chemical notes)	Macadamia oil (0)
Earthy flavor	Aromatics associated with damp soil	Roasted peanuts + skin (60)
Burnt flavor	Aromatics associated with a very dark roast, burnt starches, or carbohydrates	Dark roasted peanuts (70)
Fruity off flavor	Aromatics associated with overripe tropical fruit (associated with banana peel, dried banana, dried pineapple, and/or guava)	Banana chips (honey dipped) (50)
Sweet taste	Taste on the tongue associated with sucrose solution/Basic taste associated with sugars^1^	2% sucrose solution (20) 5% sucrose solution (50) Black Cat smooth peanut butter (40)
Salty taste	The taste on the tongue associated with sodium chloride solutions/Basic taste associated with sodium salts^2^, ^3^	0.2% sodium chloride solution (20) 0.35% sodium chloride solution (40)
Sour taste	The taste on the tongue associated with acid solutions^2^	0.05% citric acid solution (30) 0.08% citric acid solution (55) Cultured Cream Crème Fraîche (50)
Bitter taste	The taste on the tongue associated with bitter agents such as caffeine solution^2^	0.05% caffeine solution (30) 0.08% caffeine solution (60) Dark roasted peanuts (30)
*Texture* ^*7*^		
Stickiness	The force required to remove a sample that adhered to the molar teeth and to the palate	Nestlé condensed milk (10) Black Cat smooth peanut butter (80)
Thickness	The force as perceived by pressing the nut butter between the tongue and mouth	Water (0) Nestlé condensed milk (20) Black Cat smooth peanut butter (90)
Graininess	The amount of pieces of the nut perceived in the mouth	Black Cat smooth peanut butter (0) Raw macadamia paste (80) Roasted macadamia paste (60)
Oily mouthcoating	The amount of residual oil perceived in the mouth after the sample is expectorated/swallowed	Black Cat smooth peanut butter (0) Macadamia oil (40)

^1^ Adapted from Eker et al. (2023).

^2^ Adapted from Gama and Adhikari (2019).

^3^ Adapted from Lima et al. (2012).

^4^ Adapted from Nyamakwere et al. (2021).

^5^ Adapted from Romeo‐Arroyo et al. (2023).

^6^ Refer to Table 3 for preparation of reference standards.

^7^ Intensities were scored on an unstructured 100‐point line scale with 0 and 100 represented by the extremes with anchor words ‘none’ and ‘prominent’ respectively.

^8^ A reference standard intensity range is given, as a second batch of the reference standard had to be produced during the panel training stage.

The study followed a completely randomized design (Lawless and Heymann 2010). Consequently, precautions against serving order bias were taken by randomizing and counterbalancing the serving order for each panelist. One replicate of each treatment group (Table 1) was served to each panelist simultaneously in each session. The samples were blind‐coded with a random three‐digit code. The panelists were instructed to evaluate the samples in the presented order, from left to right, and palate cleansing was compulsory between samples.

Aroma attributes were assessed first, and panelists were instructed to close the lid of the sample cup frequently as the volatile aromas were sensitive and dissipated quickly. The aroma was followed by appearance attributes, which were assessed on the back of a teaspoon and compared with the customized color gradient chart (Table 2). Thereafter, panelists evaluated the palate attributes (flavor and taste). The textures were assessed last. The re‐tasting of a sample was allowed due to the extensive nature of the questionnaire; however, no re‐tasting was allowed once the evaluation of a specific sample was completed. Sensory data was captured using Compusense software (Compusense Inc., Guelph, Canada).

### 2.5 Statistical Analyses

Pre‐processing of sensory data involved testing for panel reliability using a model that includes panelist and sample effects (Næs et al. 2010). Where the Shapiro–Wilk test indicated significant deviation from normality (*p* < 0.050), and the standardized residual for an observation deviated by more than three standard deviations from the model value, outliers were removed. Following confirmation of panel reliability and normality, statistical analyses were conducted on means over panelists.

Sensory and instrumental data were subjected to univariate Analysis of Variance (ANOVA) according to the completely randomized experimental design to test treatment effects. Univariate analyses were performed using SAS software (Version 9.4, SAS Institute Inc., Cary, USA). Fisher's least significant difference (LSD) was calculated at the 5% level to compare treatment means for significant effects. A probability level of 5% was considered significant. Multivariate principal component analysis (PCA), employing the correlation matrix, was performed to clarify the association among treatments, sensory profiles, and physicochemical data using XLStat (Lumivero, 2024; XLSTAT statistical and data analysis solution). The relationship between the physicochemical and sensory attributes of nut butters was investigated through a Pearson correlation test with correlation coefficients (r).

## 3 Results and Discussion

### 3.1 Physicochemical Parameters of Macadamia Nut Butters

#### 3.1.1 Viscosity

The viscosity and overall rheological behavior of macadamia nut butter indicate product mouthfeel and quality (Shuai et al. 2023). Style 4 butter, produced from immature kernels, was the most viscous (Table 4). This may have been due to the reduced oil in the immature kernels (Wall 2013), which created a thicker product. The mean viscosity of Style 4 macadamia nut butter in this study (11939.26 mPas) was higher than walnut butter (2704 mPas) and lower than tahini (18,266 mPas) (De Jonge et al. 2023). Style 4 macadamia nut butter differed (*p* = 0.0018) from the Commercial control and the other nut butter treatments. This indicates that immature nuts, with lower oil content, produce thicker macadamia nut butters. The implication for nut butter manufacturers would be increased grinding time for immature nut butters.

**TABLE 4:** Physicochemical attributes of the macadamia butter treatments (means ± standard deviation).

Attributes		*p*	Treatments^1^				
			Commercial control	Style 1	Style 2	Style 4	Style 8
Viscosity (mPas)^2^		0.0018	6483.03^b^ ± 2836.46	6751.09^b^ ± 2358.69	5833.55^b^ ± 2660.74	11939.26^a^ ± 4518.13	6773.08^b^ ± 913.23
Oil separation (%)		< 0.0001	20.04^b^ ± 1.80	19.58^b^ ± 1.27	24.49^a^ ± 2.69	18.55^b^ ± 1.44	18.74^b^ ± 0.77
Total fat content (%)		< 0.0001	67.55^ab^ ± 4.14	67.56^ab^ ± 2.84	66.07^b^ ± 4.27	56.32^c^ ± 4.26	69.97^a^ ± 0.81
Color index^3^	L*	< 0.0001	47.927^c^ ± 2.02	57.775^a^ ± 2.10	54.587^b^ ^4^ ± 2.29	50.271^c^ ± 3.62	53.409^b^ ^4^ ± 1.54
	a*	< 0.0001	3.709^b^ ± 0.94	1.883^c^ ± 1.01	4.307^b^ ^4^ ± 0.94	6.484^a^ ± 1.06	1.809^c4^ ± 0.55
	b*	< 0.0001	20.326^c^ ± 1.24	20.830^c^ ± 1.51	22.650^b^ ^4^ ± 0.98	24.387^a^ ± 0.68	19.845^c^ ^4^ ± 1.06
	h°	< 0.0001	10.263^b^ ± 2.13	5.047^c^ ± 2.30	10.725^b^ ^4^ ± 2.16	14.870^a^ ± 2.32	5.155^c^ ^4^ ± 1.26
	C*	< 0.0001	20.675^c^ ± 1.35	20.931^c^ ± 1.59	23.070^b^ ^4^ ± 1.07	25.252^a^ ± 0.73	19.932^c^ ^4^ ± 1.10
	BI	< 0.0001	60.159^b^ ± 7.94	46.467^c^ ± 6.19	58.575^b^ ^4^ ± 5.27	74.945^a^ ± 9.26	48.061^c^ ^4^ ± 3.10

^1^ Distinct alphabetical labels were assigned to post hoc groups; a statistically significant difference (*p* < 0.050) between treatments was indicated by a different letter assigned to the corresponding attribute.

^2^ Data from 0 s to 180 s are excluded due to stirring and acclimation to temperature. Mean results from 181 s to 300 s are included.

^3^ L* = coordinate position on the lightness axis; a* = coordinate position on the green‐red axis; b* = coordinate position on the blue‐yellow axis; h = hue angle (°); C* = chroma; BI = browning index.

^4^ Outliers for one replicate of Style 8 and one replicate of Style 2 were removed from the dataset (thus, these treatments consisted of the results of 7 batches rather than 8).

The viscosity of the Commercial control and Styles 1, 2, and 8 (mean viscosity range: 5833.55–6773.08 mPas) was not different (*p* > 0.050), confirming that the grinding time was appropriate for these Styles to match this quality attribute. Furthermore, the viscosities of the Commercial control and Styles 1, 2, and 8 were closer aligned to whole macadamia butter (without stabilizer) at a shear rate of 0.2 (1/S; ca. 6000 mPas) (Shuai et al. 2024). The viscosity of Style 8 macadamia nut butter was the most consistent among treatment batches. This suggests that the quality of macadamia nut butter produced from Style 8 pieces may be well suited to a replicable product thickness. This is valuable to manufacturers who wish to produce more consistent nut butter product viscosities, especially using lower‐cost nut pieces compared to whole nuts (e.g., Style 1).

#### 3.1.2 Oil Separation

The centrifugal oil separation is a suggested indicator of the storage stability of macadamia nut butter (Shuai et al. 2024). Oil separation during storage is considered a problem within the nut butter product category (Shakerardekani et al. 2013; Zhang et al. 2019). Style 2 butters, with the longest blending time, had the highest percentage of oil separation (24.49%; Tables 1 and 5). The amount of centrifugal oil separation observed for Style 2 macadamia nut butter (despite having the highest oil separation) was similar to macadamia nut butters with 2% glyceryl monostearate, 3% beeswax, or 1% rice bran wax stabilizers (Shuai et al. 2024). However, the macadamia nut butters in the study by Shuai et al. (2024) were subjected to an extensive 65 min of grinding. Therefore, it is speculated that an increase in grinding time will increase the ‘free oils’ in the macadamia nut butter, thereby creating a more unstable product. This trend was also observed in sesame seed butter (Zhang et al. 2019). Future research should investigate whether extended blending increases oil separation during nut butter storage.

**TABLE 5:** Visual appearance related to the uniformity evaluation of treatment batches (*N* = 40) of macadamia nut butter.

Treatment	Visual uniformity in the appearance of samples
Commercial control	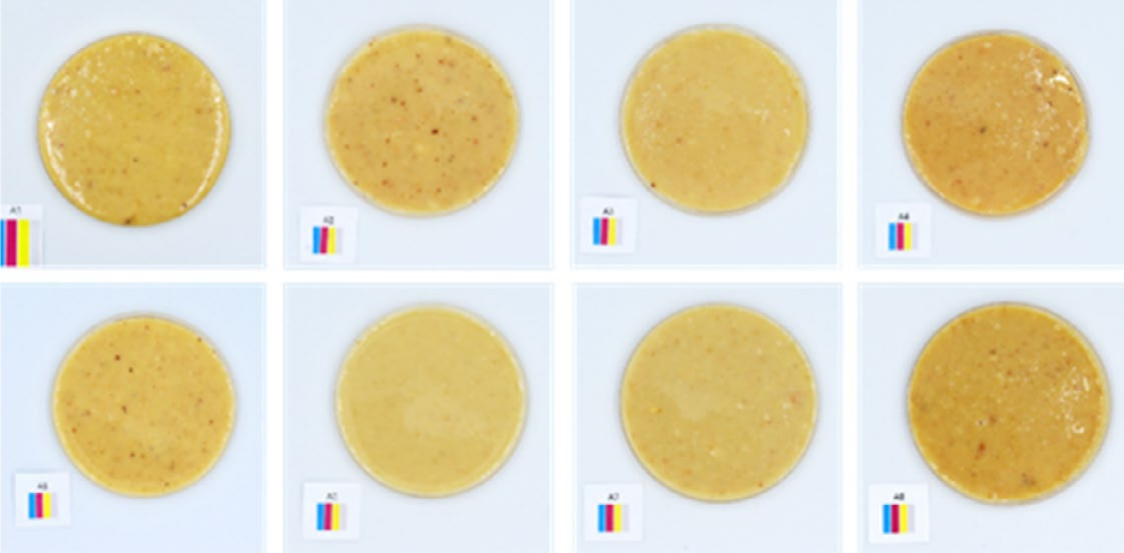
Style 1	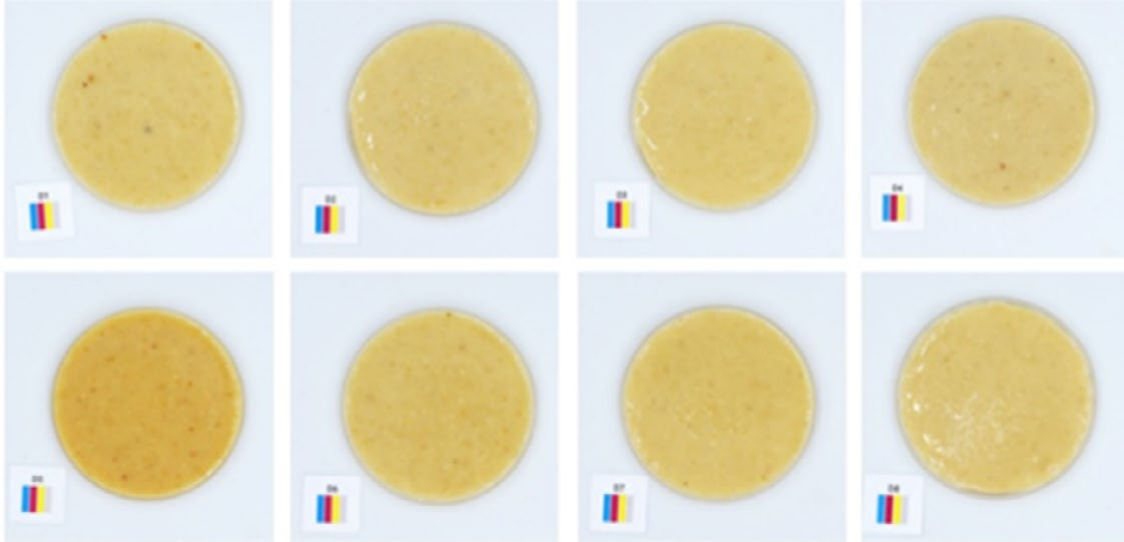
Style 2	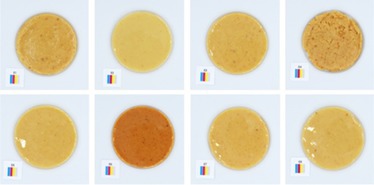
Style 4	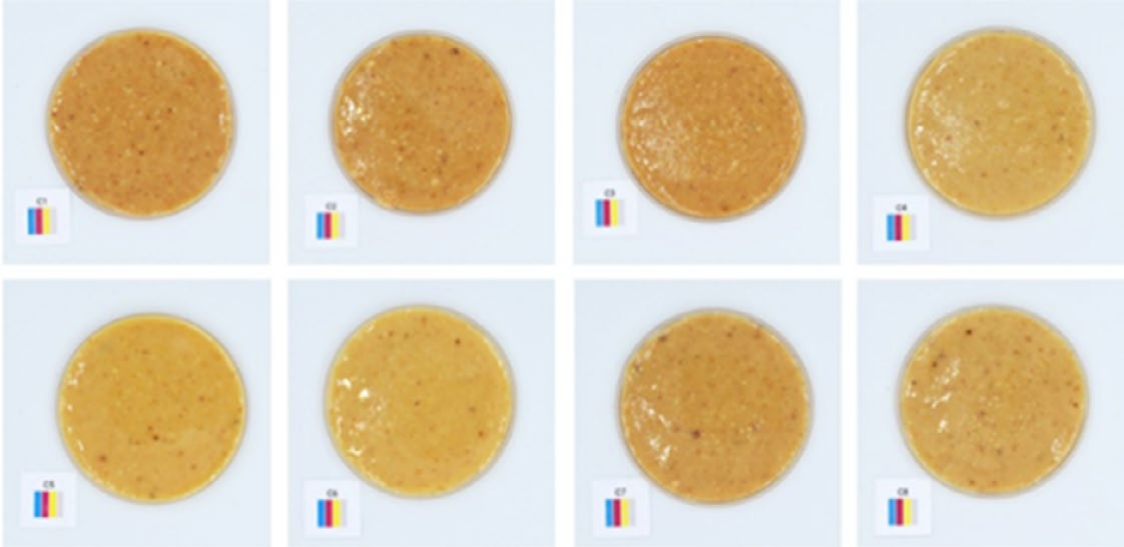
Style 8	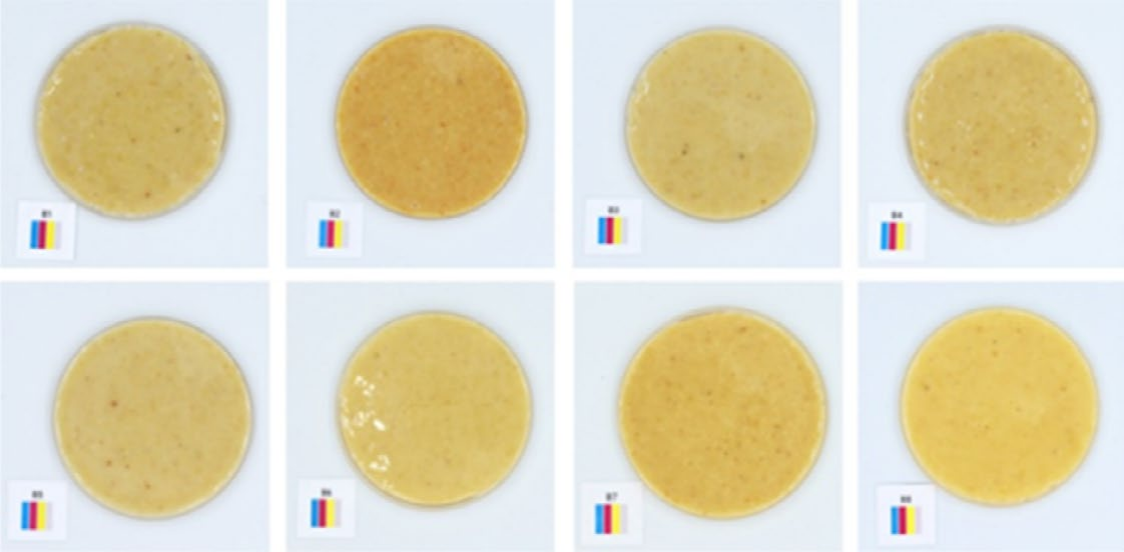

#### 3.1.3 Color

The color of the macadamia nut butter samples provides insight into the degree of roasting and is an essential appearance consideration for consumer acceptance of the product (Shuai et al. 2024; Yoon et al. 2024). Overall, macadamia nut butter showed an L* intensity (lightness) in the range of 47.93–57.78, a slightly red a*‐value in the range of 1.81–6.84, and a yellow b*‐value in the range of 19.85–23.07 (Table 4). Although the experimental treatments were all roasted for 20 min at 180°C, there were significant differences in the color parameters between treatments. The lightest (*p* < 0.001) samples were observed for Style 1 butter, potentially due to the least surface area of the whole nuts exposed to roasting conditions, and the darkest samples were observed for the Commercial control and Style 4 butters. The L* values for macadamia nut butters in this study are lower (indicating darker‐colored butter) than the results reported by Shuai et al. 2024 for unroasted macadamia nut butter (L* = 66.81–71.87). This can be expected due to the influence of roasting on imparting darker pigments. However, the nut butter L* values in the current study were lighter than roasted pecan butter (41.3–44.0; Wagener and Kerr 2018), pistachio butter (36.72; De Jonge et al. 2023), and walnut butter (41.38; De Jonge et al. 2023).

The hue angle (h°) and chroma (C*) values were highest (*p* < 0.001) for Style 4 butters, making the color of these butters more saturated and vivid compared to the other samples. The lowest hue angle and chroma values were observed for Style 8 and Style 1 butters. Both produced from more mature nuts, with similar (*p* > 0.050) nut butter hues with less intense color saturation. The mean chroma values of the butters in this study are compared with those reported for unroasted macadamia nut butter (C* = 20.36–23.14), roasted cashew nut spread (C* = 22.99), roasted cashew nut paste (C* = 21.54), roasted hazelnuts (C* = 20.15), and raw pecan nuts (C* = 21.23) (Mohammed et al. 2023; Olatidoye et al. 2017; Pham et al. 2023; Saklar et al. 2001; Shuai et al. 2024). However, the mean chroma values are higher than those for unroasted macadamia nut butter without stabilizers (C* = 16.80; Shuai et al. 2024). Furthermore, the hue results were higher than those reported for unroasted macadamia nut butters (−1.57–1.57; Shuai et al. 2024). This suggests that macadamia nut butter in the current study was less vivid in color than nut butters from other tree nut species. Furthermore, roasting enhances the color vibrancy of macadamia nut butter compared to its raw counterpart.

The most intense (*p* < 0.001) level of browning, demonstrated by BI, was observed for Style 4 butters. Roasted walnut butter (BI = 60.82–60.99) was similar to the Commercial control butters' BI (De Jonge et al. 2023; Leahu et al. 2022). The higher BI values of Style 2 and Style 4 butters may be related to the immaturity and higher reducing sugar content of these nut styles (Wall 2013). Therefore, increased roasting and BI of macadamia nuts, due to immaturity, can be observed in macadamia nut butters produced from these varieties. Conversely, the lowest BI was observed for the mature nut Style 8 and Style 1 butters.

#### 3.1.4 Uniformity

The uniformity in appearance of the product was deemed a necessary consideration for product consistency. When considering the color results alongside the uniformity, it was noted that the darker color was typical of Style 4 butter (Table 5). Although uniformity could not be quantified directly and objectively, the most uniform appearance was observed for Style 1 butter, and the least uniform appearance was observed for Style 2 butter. This would suggest that the macadamia nuts used to produce Style 2 butter (Table 1) are not well suited to producing a consistent nut butter product. Manufacturers should be mindful of the broader tolerances in kernel quality when using low‐oil, immature macadamia nuts in nut butter formulations. Additionally, the higher quality macadamia nuts (i.e., Styles 1 and 8) produced more uniform butters. Nonetheless, it was evident that all nut butter treatments had a degree of deviation in product uniformity (Table 5).

#### 3.1.5 Total Fat Content

The fat content of macadamia nuts can vary based on the maturity of the kernel; however, the total fat content is expected to fall within the range of 64.0%–80.9% (Wall 2013). Style 4 macadamia nut butter (produced from immature kernels) was the only treatment found to be outside of this expected range (56.32%). Style 4 had a (*p* < 0.001) lower fat content compared to the other treatments (Table 4). The highest fat content was observed for Style 8 macadamia nut butter (69.97%). Despite Style 2 being labeled ‘low‐oil’ by the nut supplier, these nut butters were slightly lower (*p* > 0.050) in fat content than Style 1, Style 8, and the Commercial control butters. This would suggest that total fat content cannot be used as an isolated quality consideration for nut butters and should be considered alongside the maturity of the kernel.

### 3.2 Descriptive Sensory Profiles

**FIGURE 1:**
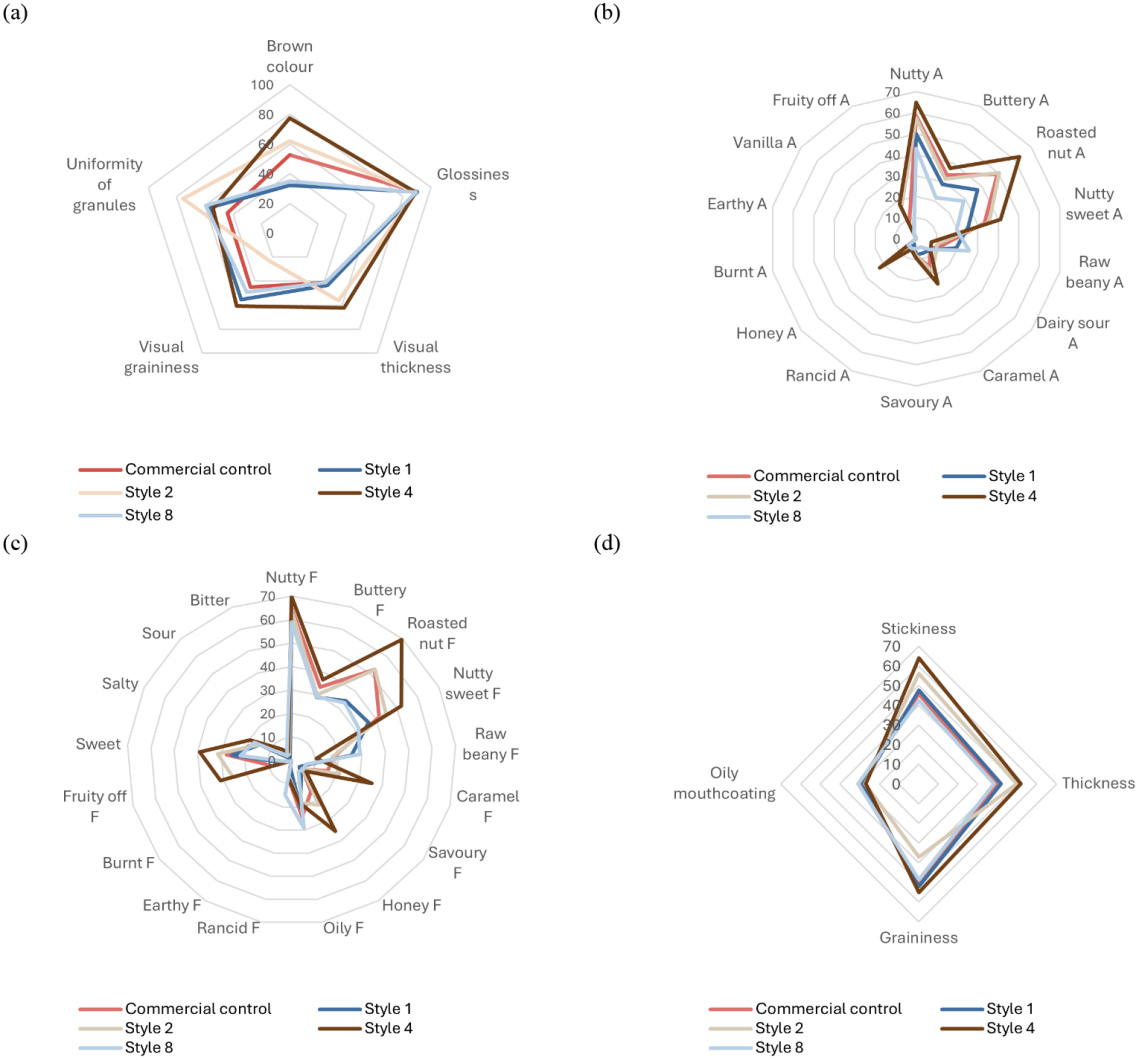
Radar charts depicting the (a) appearance, (b) aromas, (c) flavors and basic tastes, (d) and texture attributes for macadamia butter treatments. A indicates ‘aroma’ and F indicates ‘flavor’.

Macadamia nut butter produced from different kernel grades (Styles and quality) was different (*p* < 0.050) from one another (Figure 1 and Table S1), as discussed based on the physicochemical properties (section 3.1). The appearance attributes followed a trend of higher scores observed for Style 4. For instance, *brown color* (77.64; *p* < 0.001), *visual thickness* (62.37; *p* = 0.026), and *visual graininess* (60.85; *p* < 0.001) were higher for Style 4 than for the other treatments. These results correspond to the physicochemical results for Style 4, which were higher (*p* < 0.050) in color saturation (C*), BI, and viscosity (Table 4). Additionally, the lower oil content, which likely contributed to the difficulty in grinding and increased grinding time (Table 1), may have led to a higher perceived graininess in the Style 4 nut butters (Figure 1 and Table S1).

As discussed previously, immature macadamia nuts are more prone to increased browning when roasted due to higher concentrations of reducing sugars (Wall 2013). Conversely, the lowest *brown color* perception was noted for Style 8 and Style 1 butters, which were not different from one another and corresponded to the instrumental color BI results (Table 4). Although the mean sensory *visual thickness* of Style 2 butters was not significantly different from Style 4 butters, Style 2 butters had lower (*p* < 0.001) *visual graininess*. Furthermore, the most uniform granules (*uniformity of granules*) were observed in Style 2, and the least uniform granules were those of the Commercial control. This would suggest that a variety of macadamia nut qualities (i.e., oil content) and Styles are used in the Commercial control butters, creating varied particle sizes after grinding. The *glossiness* saw no significant differences between attributes (*p* = 0.749), and all nut butters were perceived as high in *glossiness* (Figure 1 and Table S1).

The aroma attributes followed a similar trend to the appearance attributes, where Style 4 had the highest intensities for nine of the 14 aroma attributes. In other words, Style 4 was highest (*p* < 0.050) in *nutty*, *buttery*, *roasted nut*, *nutty sweet*, *caramel*, *savory*, *honey*, *rancid*, and *fruity off* aroma. It also had the lowest (*p* < 0.050) intensity for *raw beany* aroma (Figure 1 and Table S1). Contrastingly, Style 8 had the lowest intensities (*p* < 0.050) for eight of the 14 aroma attributes: *nutty*, *buttery*, *roasted nut*, *nutty sweet*, *caramel*, *savory*, *honey*, and *fruity off* aroma. These trends may indicate that lower‐maturity nuts, with higher reducing sugars and susceptibility to increased roasting, were more aromatic at the chosen sample production parameters (section 4.3.2). It may also indicate that the finer and more mature nut particles (i.e., Style 8) reduced the intensity of the volatile aromatics even further. Style 1 had higher (*p* < 0.001) intensities of *nutty*, *buttery*, *roasted nut*, *nutty sweet*, and *savory* aromas than Style 8. Style 2 had the highest intensity (*p* < 0.050) of *dairy sour* aroma and shared the highest intensity of *rancid* and *fruity off* aroma with Style 4. Style 8 also shared the highest intensity of *rancid* aroma. Overall, mature varieties of macadamia nuts produced butters with lower intensity aroma profiles, whereas immature and low oil macadamia nuts produced butters with high intensities in most aroma attributes.

A honey aroma (benzeneacetaldehyde) was noted as a product of oxidation in almonds (Valdés García et al. 2021). Although not quantified in this study, this may suggest that a honey aroma could be indicative of oxidation in macadamia nut butter samples. A fruity aroma may also be indicative of oxidized nuts if associated with volatiles such as 1‐butanol, 1‐pentanol, or 1‐hexanol (Agila and Barringer 2012; Huo et al. 2024; Leal et al. 2023; Lipan et al. 2020; Liu et al. 2022; Lykomitros et al. 2016). Furthermore, caramel or otherwise “milky” aromas (associated with lactone volatiles) have been noted in oxidized almonds, cashews, and hazelnuts (Agila and Barringer 2012; Valdés García et al. 2021; Zhou et al. 2023). Therefore, oxidized (i.e., lower quality) macadamia nut butter would be expected to have stronger caramel, fruity, and honey‐like aromas. This would suggest that Style 4 macadamia butter, having the highest intensities for these attributes, may have been slightly more oxidized or associated with oxidized attributes. Neither of these instances would be desirable in the final macadamia nut butter quality. This highlights the trade‐off manufacturers face: selecting a macadamia nut style and quality (e.g., low oil) that enhances positive aroma attributes (e.g., nutty, buttery, sweet) for the same mass of nut butter (e.g., Style 4). However, this choice may also lead to higher intensities of negative attributes, such as rancid or fruity off‐aromas.

Because flavor (i.e., retronasal aroma) and aroma (i.e., orthonasal aroma) are related, and both stimulate olfactory receptors (Piombino et al. 2019), it is unsurprising that the flavor attributes mirrored the results of the aroma attributes. Style 4 had the highest flavor intensity (*p* < 0.050) for eight of the 13 flavor attributes: *nutty*, *buttery*, *roasted nut*, *nutty sweet*, *caramel*, *savory*, *honey*, and *fruity off* flavor. It also had the lowest intensities (*p* < 0.050) for *raw beany* and *oily* flavors. Enhancing the roasted nut flavor in macadamia butter may improve consumer acceptance, as roasted flavors were indicative of consumer liking in similar products, like sunflower seed butter (Vene et al. 2025).

However, for eight of the 13 flavor attributes, Style 1 and Style 8 (i.e., both made from mature nuts; Table 1) were not different (*p* > 0.050) from each other. Styles 1 and 8 shared the lowest intensities of *nutty*, *buttery*, *roasted nut*, *caramel*, *honey*, *earthy*, *burnt*, and *fruity off* flavor. Styles 1 and 8 also shared the highest intensities (*p* < 0.050) for *raw beany* flavor. Styles 1, 2, and 4 were equally the least *oily* in flavor. Two aroma and flavor attributes were found not significant (i.e., mean intensities < 5) (Joubert et al. 2024) and not different between treatments (*p* > 0.050). These were *burnt* (aroma: *p* = 0.431; flavor: *p* = 0.175) and *earthy* (aroma: *p* = 0.721; flavor: *p* = 0.704). However, these attributes were included in the ANOVA results (Table S1) for completeness.

The taste of nut butter is one of the leading motivators for its consumption (Yong et al. 2017). Basic tastes, including sweet, salty, sour, and bitter, were found to be significantly different among treatments. Style 4 butters had the highest intensities (*p* < 0.050) for *sweet*, *salty*, and *sour* tastes. Style 2 butters shared the highest intensity for the *sour* taste and were not different (*p* > 0.050) from Style 4 in *sour* taste. The highest intensity for *bitter* taste was seen for Style 8 butters, although the intensity was very low and would most likely not be perceived by the untrained palate (i.e., consumers). Style 1 butter had the lowest intensities (*p* < 0.050) for *salty*, *sour*, and *bitter* tastes. The lowest intensity for *sweet* taste was observed for Style 8 butters. Considering the increasing consumer health awareness, lower‐fat and lower‐sugar options (such as Style 4 butters) are expected to gain popularity (Vene et al. 2025). These results may serve as a guide to manufacturers who may be interested in producing a lower added‐sugar variety or even flavoring their macadamia nut butter. For example, the higher natural sweetness of the immature qualities (e.g., Style 4) may reduce the amount of added sugar required, just as the natural higher saltiness may serve as a flavor enhancer (Elias et al. 2020). However, this requires further investigation with benchmarking against flavored samples to confirm.

The textures perceived on the palate for the macadamia nut butters included *stickiness*, *thickness*, *graininess*, and *oily mouthcoating* (Table S1). As expected, based on the physicochemical results (section 3.1), the highest intensities were seen in Style 4 butters for three of the four texture attributes: *stickiness*, *thickness*, and *graininess*. However, Style 4 also had the lowest intensity rating for *oily mouthcoating*, which was shared (*p* > 0.050) with Style 2. The lowest intensities (*p* < 0.050) for *stickiness* were shared by Commercial control and Style 8 butters. These treatments also shared the lowest intensities for *thickness*.

The end of the grinding time was measured by comparing the viscosity of the sample in the lab scale blender to the final viscosity of a control sample from the industrial blender. Some of the treatments required longer grinding times than others. Furthermore, the precise grinding time for the Commercial control is unknown. It is speculated that the higher oil content in Styles 1 and 8 allowed them to blend more rapidly than that of Styles 2 and 4. This supports the difference in oil content and maturity (i.e., qualities) of the nut kernels, which play an important role in the quality of the final macadamia nut butter.

**FIGURE 2:**
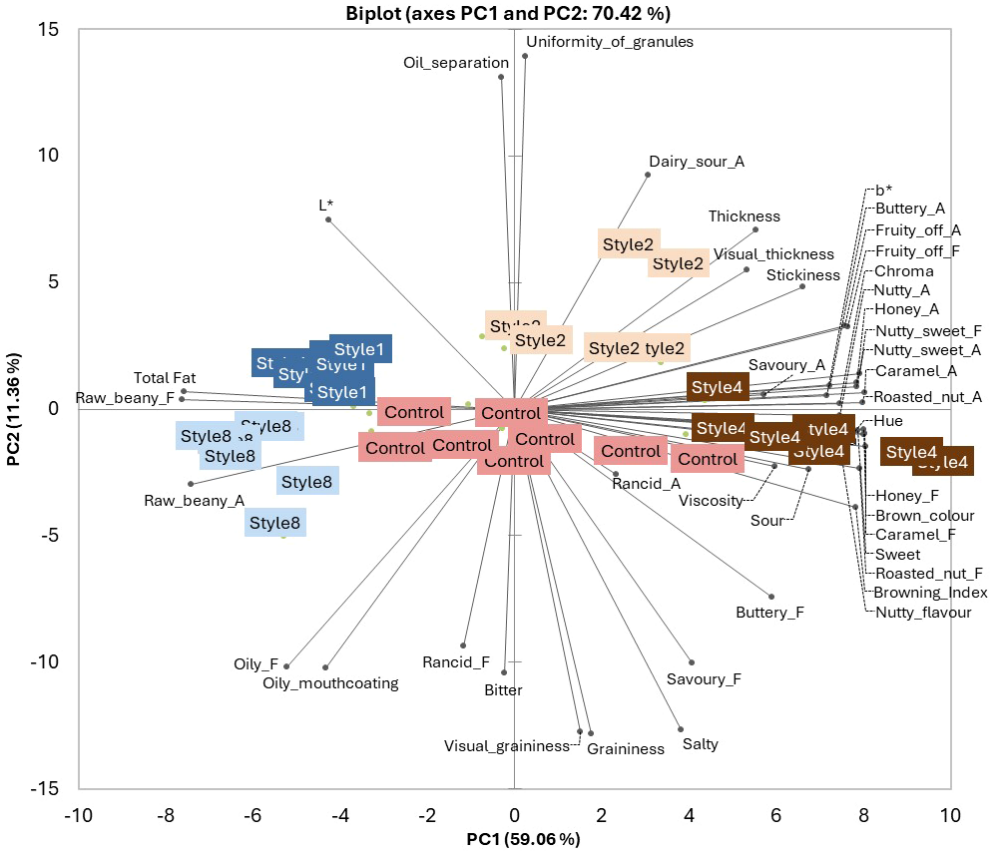
Principal components analysis (PCA) biplot depicting appearance, aroma (A), flavor (F), taste, and texture attributes of macadamia butter treatment batches. *Salty*, *sweet*, *sour*, and *bitter* all refer to basic tastes. Four of the extreme treatment batches (batch 5 of Style 1, batches 2 and 6 of Style 2, and batch 2 of Style 8) were removed to be able to interpret the chart; however, these batches were still captured in the GLM model and ANOVA. Attributes that were not significant (*p* > 0.050; that is, *burnt* aroma, *burnt* flavor, *earthy* aroma, *earthy* flavor, and *glossiness* appearance) were removed.

Principal component analysis (PCA; 70.42% explained variance; Figure 2) highlighted the trends for the different macadamia nut butter treatments and the variables (physicochemical and sensory attributes). It included the five treatments (*n* = 5), viscosity, oil separation, color attributes, total fat, and the significant sensory attributes (*n* = 34). PC1 accounted for 59.06% of the data variance, and most variables were associated with this PC. However, *uniformity of granules* and *visual graininess* (sensory appearance attributes), *dairy sour* aroma, *salty* taste, *savory* flavor, and *oily mouthcoating* and *graininess* (sensory texture attributes), as well as oil separation (instrumental measurement), were associated with PC2, which accounted for 11.36% of the data variance. *Rancid* aroma, *bitter* taste, and the L* value were associated with subsequent PC and are, therefore, not explained by Figure 2.

The PCA biplot confirms the inconsistent sensory profiles of the Commercial control batches. Furthermore, the central location of the Commercial control batches indicates no strong association with any of the variables on principal component 1 (PC1). This could be explained by some variation in product formulation between batches, inconsistency in raw material quality (which is more likely when considering the variation seen in other treatments), or inconsistency in processing parameters such as roasting and/or grinding times.

Mature kernel varieties (represented by Styles 1 and 8) produced a more replicable butter quality between batches (Figure 2) and had the highest (*p* < 0.001) total fat content (Table 4). Furthermore, the total fat content was moderately positively correlated (*p* < 0.050) to *oily* flavor (*r* = 0.665), *raw beany* aroma (r = 0.736), and *raw beany* flavor (r = 0.765). Except for *oily mouthcoating* (Figure 1 and Table S1), the textural attributes of Styles 1 and 8 nut butters were not influenced by the size of the kernels or pieces. These similarities substantiate that fine pieces (i.e., Style 8) can be incorporated into macadamia nut butter without detriment to the sensory quality of the product. This confirms the suggestion by Lima et al. (2012) that value can be added to nut pieces through their incorporation into nut butters. These findings are promising for both input cost reduction and food waste reduction.

Immature kernel and lower oil varieties (represented by Styles 2 and 4) were more inconsistent between batches. Style 2 differed from other treatments largely because of its textural profile (PC2), which may be related to the increased blending time for this treatment (Table 1). The texture profile included increased *uniformity of granules* (appearance attribute), the least *graininess* (texture attribute), and *visual graininess* (appearance attribute) (Figure 1 and Table S1). Style 4 nut butters were associated with assumed positive *sweet*, *nutty*, and *roasted* attributes, as well as a higher degree of *brown color* intensity. However, this nut butter treatment was also associated with negative sensory attributes such as *honey* aroma, *fruity off* aroma, and flavor, which have been associated with oxidation in other nut butters (Agila and Barringer 2012; Huo et al. 2024; Leal et al. 2023; Lipan et al. 2020; Liu et al. 2022; Lykomitros et al. 2016). *Honey* aroma had a strong positive correlation (*p* < 0.050) to *fruity off* aroma (*r* = 0.934) and flavor (r = 0.905). The reference standard for *fruity off* aroma used in this study required more preparation as compared to the honey reference sample (Table 2). Therefore, these results are promising, as honey could be used as a straightforward potential indicator of oxidation during macadamia nut butter quality control. Future research could confirm this link and consider the oxidation threshold linked to the presence of a *honey* aroma.

Despite each experimental treatment being roasted at consistent conditions of 180°C for 20 min, it is theorized that roasting time influences the sensory attributes of the treatments differently. Furthermore, future studies could consider the concentration of reducing sugars present in immature kernels and how this may result in the development of different volatile aroma compounds and browning pigments during the roasting process.

## 4 Conclusion

This study aimed to address the knowledge gap in the overall product quality of macadamia nut butter by evaluating butters produced from individual kernel styles and qualities. The research also provides the first sensory analysis of macadamia nut butter and developed a sensory lexicon of 40 descriptors.

Immature Style 4 kernels, with the lowest oil content, produced the most viscous butter, exhibiting higher *visual thickness*, *stickiness*, and *graininess*. These kernels also showed the most intense color (higher hue, chroma, and browning index), likely due to higher reducing sugar content. This resulted in the highest intensities for both positive and negative sensory aroma, flavor, taste, and texture attributes.

Style 2 kernels, while producing butters with comparable *brown color* intensity and *sour* taste to Style 4, required extended grinding, which led to increased oil separation in the final product. Future studies should confirm whether extended blending time for nut butters is responsible for increased oil separation during storage.

Mature, high‐oil kernels (Styles 1 and 8) produced butters with lower viscosities, color intensity, and sensory attributes, similar to the Commercial control. This suggests that kernel oil content and maturity, rather than size, most significantly impact the sensory quality of the final product. This research offers manufacturers and ingredient procurers valuable insights into the effects of macadamia nut quality and grades on the quality of processed products like nut butter. Style 8 pieces would be the more economical option for manufacturers and would reduce potential food waste.

The main limitations of this foundational investigation are the chosen four macadamia styles and the standardized roasting time. These findings highlighted the need to customize roasting conditions based on kernel maturity and oil content. Immature, low‐oil nuts may require milder roasting and longer grinding times, whereas mature, high‐oil nuts would benefit from more intense roasting and shorter grinding. Customizing these parameters could enhance product consistency and address current issues of variability in macadamia nut butter quality.

## Author Contributions

**Tarryn Amber Ohlsson:** data curation (equal), investigation (lead), methodology (equal), visualization (lead), writing – original draft (lead), writing – review and editing (supporting). **Marieta van der Rijst:** data curation (equal), formal analysis (lead), writing – review and editing (supporting). **Jeannine Marais:** conceptualization (lead), data curation (equal), formal analysis (supporting), funding acquisition (lead), investigation (supporting), methodology (equal), project administration (lead), resources (lead), supervision (lead), validation (lead), visualization (supporting), writing – original draft (supporting), writing – review and editing (lead).

## Conflicts of Interest

The authors declare no conflicts of interest.

## Supporting information


**Data S1:** fsn370773‐sup‐0001‐Supinfo01.docx.

## Data Availability

The data that support the findings of this study are available on request from the corresponding author. The data are not publicly available due to privacy or ethical restrictions.
