# Supplementary material for: Sensory Quality of Macadamia Nut Butter: Effects of Kernel Grade and Maturity
Source: Food Sci Nutr. 2025 Aug 8;13(8):e70773. doi: 10.1002/fsn3.70773 (PMC12334548; doi:10.1002/fsn3.70773)
Supplement: Supplementary file 1 — Data S1: fsn370773‐sup‐0001‐Supinfo01.docx. [file FSN3-13-e70773-s001.docx]

**Supplemental Information**


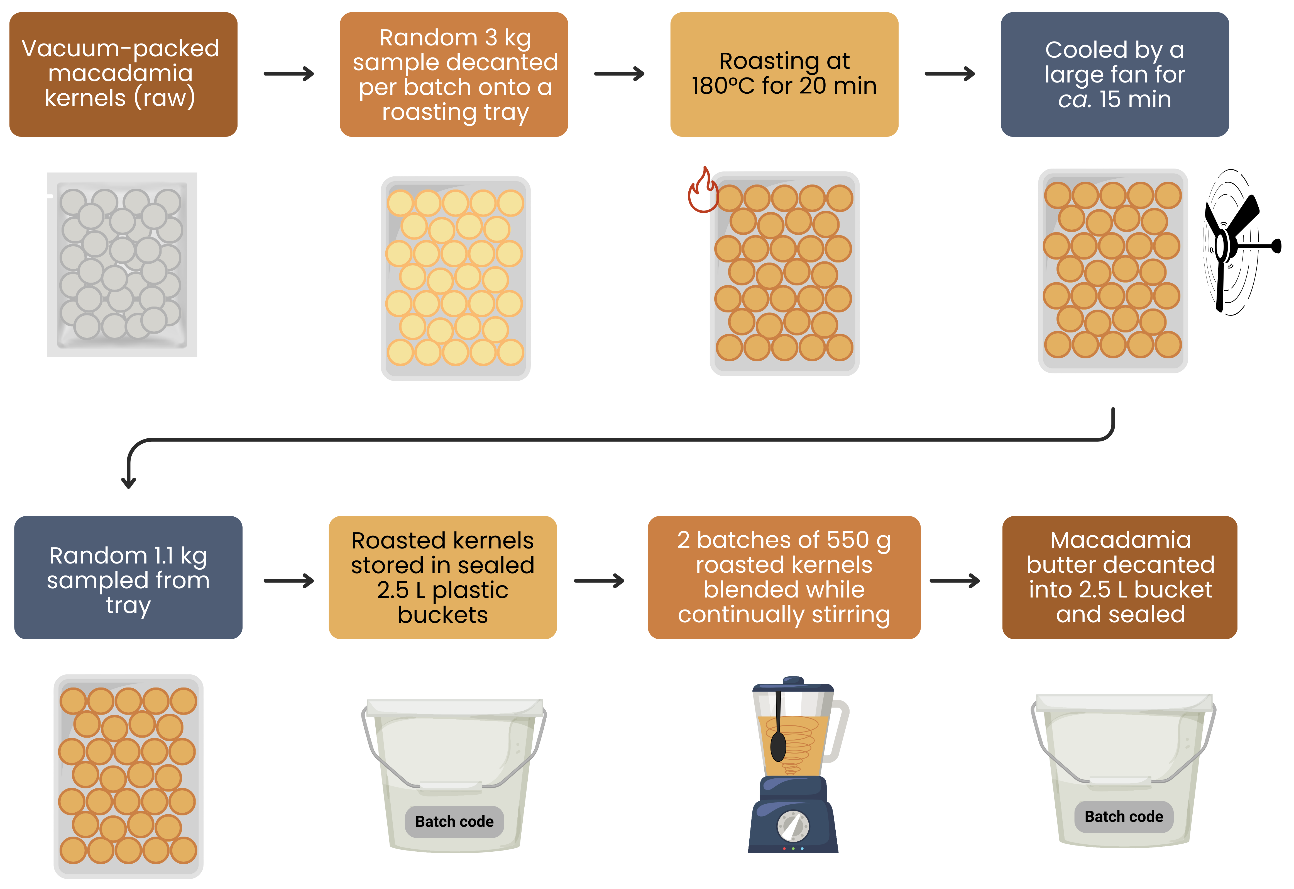


**Figure S1** Small-scale production of macadamia nut butter (images sourced from canva.com).

| Initial appearance of roasted Style 1 nuts | Paste after 20 s of grinding | Butter after 35 s  of grinding |
| --- | --- | --- |
| 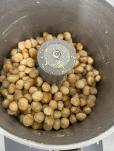 | 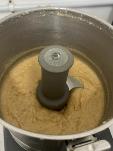 | 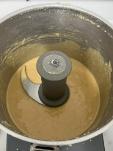 |

**Figure S2** Appearance of Style 1 macadamia nut butter throughout the grinding process.

| Initial appearance of roasted Style 2 nuts | Paste after 30 s  of grinding | Paste after 60 s  of grinding | Paste after 90 s  of grinding | Butter after 150 s of grinding |
| --- | --- | --- | --- | --- |
| 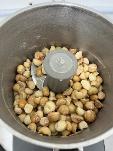 | 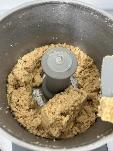 | 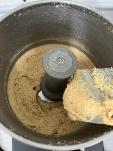 | 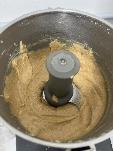 | 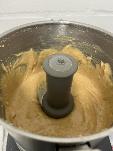 |

**Figure S3** Appearance of Style 2 macadamia nut butter throughout the grinding process.

| Initial appearance of roasted Style 4 nuts | Paste after 30 s  of grinding | Paste after 60 s  of grinding | Butter after 80 s  of grinding |
| --- | --- | --- | --- |
| 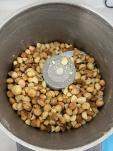 | 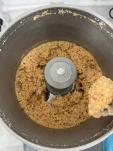 | 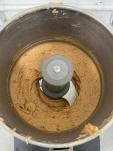 | 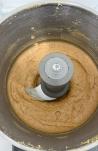 |

**Figure S4** Appearance of Style 4 macadamia nut butter throughout the grinding process.

| Initial appearance of roasted  Style 8 nuts | Paste after 20 s  of grinding | Butter after 42 s  of grinding |
| --- | --- | --- |
| 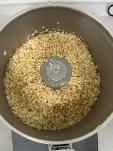 | 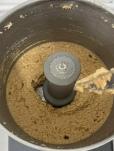 | 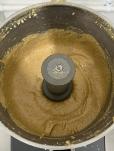 |

**Figure S5** Appearance of Style 8 macadamia nut butter throughout the grinding process.


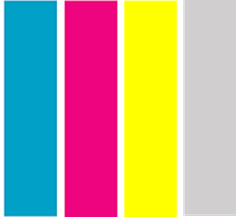


**Figure S6** Color standard containing cyan (RGB color code: 1, 160, 198), magenta (RGB color code: 239, 4, 127), yellow (RGB color: 255, 255, 0), and grey (RGB color code: 208, 206, 206) that was used in each sample label during photography [This figure requires color for accurate representation in print].

**Table S1** Intensities (means ± standard deviation) of sensory aroma, appearance, flavor, taste, and texture attributes

|  |  | **Treatments^1^** | | | | |
| --- | --- | --- | --- | --- | --- | --- |
| **Attributes** | ***p*-value^2^** | **Commercial control** | **Style 1** | **Style 2** | **Style 4** | **Style 8** |
| **Appearance** |  |  |  |  |  |  |
| Brown color | **< 0.0001** | 52.73^c^ ± 11.3 | 32.26^d^ ± 2.6 | 62.04^b^ ± 5.7 | 77.64^a^ ± 8.2 | 35.11^d^ ± 7.2 |
| Glossiness | 0.7487 | 90.06^a^ ± 1.6 | 90.44^a^ ± 0.6 | 89.28^a^ ± 3.5 | 89.04^a^ ± 2.9 | 89.47^a^ ± 1.7 |
| Visual thickness | **0.0258** | 41.30^b^ ± 6.8 | 43.35^b^ ± 11.8 | 56.18^ab^ ± 28.7 | 62.37^a^ ± 14.6 | 41.31^b^ ± 4.5 |
| Visual graininess | **< 0.0001** | 44.95^c^ ± 6.8 | 55.33^ab^ ± 7.8 | 23.20^d^ ± 11.0 | 60.85^a^ ± 11.5 | 49.16^bc^ ± 3.4 |
| Uniformity of granules | **< 0.0001** | 44.19^c^ ± 3.0 | 56.02^b^ ± 2.6 | 75.47^a^ ± 10.7 | 54.21^b^ ± 4.0 | 59.57^b^ ± 3.6 |
| **Aroma** |  |  |  |  |  |  |
| Nutty aroma | **< 0.0001** | 58.58^b^ ± 4.9 | 50.07^c^ ± 3.1 | 57.42^b^ ± 2.2 | 64.92^a^ ± 3.8 | 43.05^d^ ± 4.4 |
| Buttery aroma | **< 0.0001** | 33.60^b^ ± 2.4 | 28.79^c^ ± 1.5 | 31.77^b^ ± 2.5 | 37.31^a^ ± 3.4 | 21.58^d^ ± 2.3 |
| Roasted nut aroma | **< 0.0001** | 49.16^b^ ± 7.1 | 37.16^c^ ± 2.4 | 50.51^b^ ± 4.7 | 62.74^a^ ± 5.6 | 28.80^d^ ± 3.5 |
| Nutty sweet aroma | **< 0.0001** | 33.23^b^ ± 5.4 | 25.39^c^ ± 2.3 | 35.48^b^ ± 2.9 | 41.08^a^ ± 2.3 | 19.72^d^ ± 3.0 |
| Raw beany aroma | **< 0.0001** | 13.11^c^ ± 5.4 | 19.54^b^ ± 2.9 | 10.36^cd^ ± 3.6 | 7.30^d^ ± 3.1 | 25.76^a^ ± 2.4 |
| Dairy sour aroma | **0.0024** | 8.19^b^ ± 1.6 | 8. 48^b^ ± 1.2 | 11.83^a^ ± 1.4 | 8.66^b^ ± 2.1 | 7.97^b^ ± 2.1 |
| Caramel aroma | **< 0.0001** | 14.75^b^ ± 4.2 | 7.59^c^ ± 2.2 | 17.45^b^ ± 3.6 | 23.85^a^ ± 3.4 | 4.32^c^ ± 1.9 |
| Savory aroma | **< 0.0001** | 8.05^ab^ ± 0.9 | 7.57^b^ ± 1.5 | 7.78^ab^ ± 1.1 | 9.12^a^ ± 1.6 | 4.88^c^ ± 1.1 |
| Rancid aroma | **0.0207** | 3.89^ab^ ± 1.4 | 2.52^b^ ± 1.8 | 5.10^a^ ± 2.1 | 5.52^a^ ± 1.8 | 5.02^a^ ± 1.9 |
| Honey aroma | **< 0.0001** | 13.09^b^ ± 3.9 | 6.64^c^ ± 2.0 | 16.69^b^ ± 3.3 | 22.19^a^ ± 4.3 | 4.95^c^ ± 1.9 |
| Burnt aroma | 0.4309 | 0.02^a^ ± 0.0 | 0.02^a^ ± 0.0 | 0.01^a^ ± 0.0 | 0.02^a^ ± 0.0 | 0.02^a^ ± 0.0 |
| Earthy aroma | **0.0379** | 0.00^c^ ± 0.0 | 0.01^bc^ ± 0.0 | 0.01^abc^ ± 0.0 | 0.02^ab^ ± 0.0 | 0.02^a^ ± 0.0 |
| Vanilla aroma | 0.7208 | 0.02^a^ ± 0.0 | 0.04^a^ ± 0.0 | 0.03^a^ ± 0.0 | 0.03^a^ ± 0.0 | 0.02^a^ ± 0.0 |
| Fruity off aroma | **< 0.0001** | 7.90^b^ ± 4.9 | 2.76^c^ ± 0.6 | 16.53^a^ ± 4.1 | 18.39^a^ ± 4.5 | 2.49^c^ ± 1.8 |
| **Flavor** |  |  |  |  |  |  |
| Nutty flavor | **< 0.0001** | 64.93^b^ ± 3.0 | 60.95^c^ ± 2.8 | 64.21^b^ ± 3.3 | 69.89^a^ ± 2.1 | 58.95^c^ ± 2.7 |
| Buttery flavor | **< 0.0001** | 33.64^b^ ± 2.3 | 29.03^c^ ± 3.6 | 29.89^c^ ± 2.9 | 36.96^a^ ± 1.6 | 29.12^c^ ± 3.4 |
| Roasted nut flavor | **< 0.0001** | 52.31^b^ ± 9.1 | 34.28^c^ ± 4.1 | 52.31^b^ ± 8.9 | 69.58^a^ ± 2.9 | 33.40^c^ ± 3.5 |
| Nutty sweet flavor | **< 0.0001** | 41.55^b^ ± 5.30 | 36.49^c^ ± 1.5 | 44.78^b^ ± 3.8 | 52.00^a^ ± 2.3 | 31.63^d^ ± 3.5 |
| Raw beany flavor | **< 0.0001** | 18.07^b^ ± 4.5 | 25.57^a^ ± 3.3 | 17.21^b^ ± 5.1 | 10.66^c^ ± 1.7 | 29.17^a^ ± 1.9 |
| Caramel flavor | **< 0.0001** | 15.80^c^ ± 6.3 | 6.77^d^ ± 1.8 | 21.28^b^ ± 5.5 | 35.37^a^ ± 3.4 | 6.08^d^ ± 1.8 |
| Savory flavor | **0.0027** | 5.90^b^ ± 1.2 | 4.21^c^ ± 1.2 | 5.55^bc^ ± 0.8 | 7.58^a^ ± 1.4 | 6.29^ab^ ± 2.3 |
| Honey flavor | **< 0.0001** | 15.57^c^ ± 6.8 | 7.87^d^ ± 3.4 | 22.28^b^ ± 5.2 | 35.34^a^ ± 2.8 | 5.68^d^ ± 0.8 |
| Oily flavor | **< 0.0001** | 24.39^b^ ± 3.6 | 20.10^c^ ± 3.03 | 18.10^c^ ± 4.0 | 18.28^c^ ± 2.67 | 29.15^a^ ± 3.2 |
| Rancid flavor | **0.0012** | 7.57^b^ ± 4.7 | 2.90^b^ ± 0.5 | 6.71^b^ ± 1.9 | 7.53^b^ ± 1.9 | 14.83^a^ ± 9.0 |
| Earthy flavor | 0.7041 | 0.04^a^ ± 0.0 | 0.06^a^ ± 0.0 | 0.06^a^ ± 0.0 | 0.05^a^ ± 0.0 | 0.06^a^ ± 0.0 |
| Burnt flavor | 0.1748 | 0.02^ab^ ± 0.0 | 0.03^ab^ ± 0.0 | 0.01^b^ ± 0.0 | 0.03^a^ ± 0.0 | 0.03^ab^ ± 0.0 |
| Fruity off flavor | **< 0.0001** | 8.43^c^ ± 5.1 | 2.98^d^ ± 1.6 | 25.85^b^ ± 4.2 | 31.33^a^ ± 3.5 | 1.78^d^ ± 1.1 |
| **Taste** |  |  |  |  |  |  |
| Sweet taste | **< 0.0001** | 27.70^c^ ± 4.6 | 25.47^c^ ± 1.2 | 31.43^b^ ± 3.9 | 39.08^a^ ± 2.6 | 21.89^d^ ± 1.3 |
| Salty taste | **0.0006** | 16.53^bc^ ± 1.8 | 15.13^c^ ± 1.0 | 15.10^c^ ± 1.4 | 19.62^a^ ± 2.1 | 17.64^ab^ ± 3.1 |
| Sour taste | **< 0.0001** | 3.32^b^ ± 2.0 | 1.20^c^ ± 0.7 | 5.42^a^ ± 1.2 | 6.89^a^ ± 1.1 | 2.60^bc^ ± 1.7 |
| Bitter taste | **0.0004** | 2.60^bc^ ± 0.9 | 1.40^c^ ± 0.8 | 2.80^bc^ ± 1.6 | 3.83^b^ ± 1.0 | 5.61^a^ ± 2.9 |
| **Texture** |  |  |  |  |  |  |
| Stickiness | **< 0.0001** | 45.66^c^ ± 7.3 | 47.57^bc^ ± 7.3 | 56.05^ab^ ± 13.63 | 64.10^a^ ± 6.5 | 42.53^c^ ± 3.9 |
| Thickness | **0.0077** | 39.36^c^ ± 4.3 | 41.78^bc^ ± 6.5 | 49.66^ab^ ± 15.6 | 51.80^a^ ± 7.7 | 38.24^c^ ± 2.4 |
| Graininess | **< 0.0001** | 50.05^b^ ± 3.9 | 52.12^ab^ ± 4.2 | 37.14^c^ ± 3.6 | 55.09^a^ ± 4.8 | 48.68^b^ ± 2.0 |
| Oily mouthcoating | **0.0033** | 30.24^ab^ ± 1.0 | 28.15^bc^ ± 2.5 | 27.28^c^ ± 3.4 | 26.94^c^ ± 1.8 | 30.92^a^ ± 1.9 |

^1^Distinct alphabetical labels were assigned to post-hoc groups; a statistically significant difference (*p* < 0.050) between treatments was indicated by a different letter assigned to the corresponding attribute.

^2^ Significant p-values (*p* < 0.050) are indicated in bold.
